# Collaborative PrEP Implementation Strategies for Latino Men Who have Sex with Men: A Health Center-Community Consensus Process

**DOI:** 10.1007/s10900-023-01266-w

**Published:** 2023-08-07

**Authors:** Jonathan Ross, Gabriela S. Betancourt, Elí A. Andrade, Augustus Klein, Lissette Marrero, Gustavo A. Morales, Sage Rivera, Dana L. Watnick, Viraj V. Patel

**Affiliations:** 1 Division of General Internal Medicine, Department of Medicine, Albert Einstein College of Medicine and Montefiore Medical Center, 3300 Kossuth Avenue, Bronx, NY 10467 USA; 2 Latino Commission on AIDS, New York, NY USA; 3 Hunter Alliance for Research and Translation, Hunter College, New York, NY USA; 4 Voces Latinas Inc, Jackson Heights, NY USA; 5 Destination Tomorrow, Bronx, NY USA; 6 Department of Pediatrics, Albert Einstein College of Medicine, Bronx, NY USA

**Keywords:** HIV, Pre-exposure prophylaxis, Latino, Community-based organization, Delphi technique

## Abstract

Community-based organizations (CBOs) deliver services in culturally-responsive ways, and could effectively partner with health centers to deliver HIV pre-exposure prophylaxis (PrEP) to Latino men who have sex with men (LMSM). However, few such models exist. We conducted a planning study in collaboration with three CBOs serving LMSM to identify optimal PrEP delivery strategies for health centers and CBOs to implement jointly. We established a Community Expert Panel (CEP) of eight client-facing CBO and health center staff. Over 6 months, the panel met monthly to identify collaborative strategies for PrEP delivery, using a modified Delphi method consisting of the following steps: (1) brainstorming strategies; (2) rating strategies on acceptability, appropriateness and feasibility; (3) review of data from qualitative focus group discussions with CBO clients; and (4) final strategy selection. The panel initially identified 25 potential strategies spread across three categories: improving communication between health centers and CBOs; using low-barrier PrEP options (e.g. telemedicine), and developing locally-relevant, culturally-sensitive outreach materials. Focus groups with CBO clients highlighted a desire for flexible options for PrEP-related care and emphasized trust in CBOs. The final package of strategies consisted of: (1) a web-based referral tool; (2) telemedicine appointments; (3) geographically-convenient options for lab specimen collection; (4) tailored print and social media; and (5) regular coaching sessions with CBO staff. Through a community-engaged process, we identified a package of PrEP delivery strategies that CBOs and health centers can implement in partnership, which have the potential to overcome barriers to PrEP for LMSM.

**Supplementary Information:**

The online version contains supplementary material available at 10.1007/s10900-023-01266-w.

## Introduction

Latino men who have sex with men (LMSM) are disproportionately impacted by HIV and incidence in this group continues to increase [1]. Pre-exposure prophylaxis (PrEP) is highly effective at preventing HIV [2, 3], is recommended for all people at substantial risk of HIV [4], and is widely available. Nonetheless, despite interest in and willingness to use it, less than one-third of eligible LMSM are accessing PrEP [5–8].

Unique barriers impact LMSM’s access to PrEP. These include factors such as HIV stigma, medical mistrust, and discrimination related to sexual identity, race and immigration status [9–12]; as well as structural barriers including lack of health insurance, limited English proficiency, and immigration enforcement [13–16]. For many LMSM, the process of obtaining PrEP is complex, uncomfortable, and may not address their needs. Because PrEP is largely available in traditional healthcare settings, the above factors act synergistically to deter many LMSM from accessing it.

There is consensus that community-based approaches are necessary to increase PrEP uptake generally and among LMSM [17, 18]. Engaging communities in health program development elevates the relevance of programming to community members and, especially in traditionally marginalized groups, can meaningfully reduce health disparities [19, 20]. Community-based organizations (CBOs) deliver services that address the needs of LMSM and are effective settings for HIV testing, raising PrEP awareness, and linkage to PrEP [21, 22]. However, CBOs are infrequently included in designing PrEP delivery systems. Shared decision-making between CBOs and health centers with respect to planning, implementing and evaluating service delivery could effectively overcome barriers faced by LMSM, yet such approaches have not been widely used.

As part of ongoing HIV prevention efforts, our research group—consisting of academic investigators at Albert Einstein College of Medicine and a community investigator working at the Latino Commission on AIDS (LCOA) at the time of the study—has been collaborating with the Montefiore Prevention Program (MPP, an HIV prevention program supporting sexual health and PrEP implementation in a network of primary care centers in Bronx, NY) and three CBOs in New York City (NYC) providing services to LMSM. Beginning in late 2020, we partnered with one of these CBOs, LCOA, to develop and pilot CBO-PrEP. This telehealth-based model includes hand-in-hand PrEP navigation conducted by CBO and MPP staff as well as flexible, patient-driven options for laboratory testing and PrEP prescribing. We found this model was feasible and acceptable to clients and staff. However, we encountered implementation barriers including lack of familiarity with telehealth, uncertainty around co-navigation workflows, logistical challenges related to insurance and obtaining diagnostic testing, and hesitancy about whether this model would overcome clients’ competing demands or medical mistrust [23, 24].

Therefore, for the current study, we sought to obtain further input from community stakeholders to enhance the CBO-PrEP program. Our objective was to identify approaches for health centers and CBOs to partner in delivering PrEP in ways that meet the needs and priorities of LMSM in NYC.

## Methods

### Setting

This current study was conducted in NYC, an epicenter of the HIV epidemic, in partnership with MPP and three CBOs based in NYC: LCOA, Voces Latinas (VL) and Destination Tomorrow (DT). We deliberately included representatives from these four institutions to reflect the diversity in NYC’s LMSM communities and the organizations delivering services to them. The three CBOs all provide social and health-related services to their clients, including HIV testing and PrEP referrals, but serve communities that are diverse in terms of geography, ethnicity, gender, sexual orientation, and time in the U.S. LCOA is a national advocacy, capacity building and service organization dedicated to addressing the impact of HIV/AIDS in Latino communities in the United States, and runs the Oasis Community Pride Center, a drop-in center in Manhattan that provides sex-positive services for LGBTQ persons. VL provides HIV prevention, mental health and insurance services to a predominantly new Latino immigrant community in Jackson Heights, Queens, including street outreach, HIV testing and navigation to PrEP. DT, the ‘Bronx LGBTQ + center,’ provides services focusing on HIV prevention, housing, mental health and syringe access in its community drop-in space, focusing on youth, transgender persons, and people who are unstably housed. Montefiore Medical Center is a major academic medical center based in the Bronx, one of NYC’s boroughs, where HIV prevalence is 2.2% and Latinos constitute > 50% of the 1.4 million inhabitants. MPP, Montefiore’s community and primary care-based HIV prevention program, focuses on outreach to sexual minority men and supports PrEP implementation in a network of over 20 primary care health centers in the Bronx [25].

### Consolidated Framework for Implementation Research

The Consolidated Framework for Implementation Research (CFIR) is an implementation framework incorporating domains and constructs that may impact implementation of interventions in various settings, structured across five key domains [26]. To understand how individual, organizational and broader contextual factors could impact potential PrEP implementation strategies, we used the CFIR as a framework to guide community expert panel discussions as well as to develop the focus group guide and analyze qualitative data. We examined perspectives on PrEP-related service delivery within the following CFIR domains: interventions (e.g., relative advantage of telemedicine over in-person appointments), outer setting (e.g., PrEP-related policies in NYC, typical approaches to PrEP navigation at CBOs), inner setting (e.g., philosophy and culture of MPP and CBOs around service delivery, partnering with academic institutions), individuals involved (e.g., PrEP-related knowledge of CBO staff), and the process of implementation (e.g., how CBOs typically plan and evaluate new initiatives).

### Community Expert Panel and Modified Delphi Process

We met with leadership at MPP, LCOA, VL, and DT to identify eight individuals (two from each organization who were client-facing staff with clinical or service provision roles and who were willing to participate in monthly meetings. We contacted each individual by email to describe the process and invite participation as a panel member. The community expert panel met virtually using Zoom once a month for 2–3 hours. The study was approved by the Institutional Review Board of the Albert Einstein College of Medicine (2021-13221); prior to the first session, panelists provided written informed consent.

We utilized a modified Delphi approach—a structured, iterative, mixed-methods process of collecting and summarizing opinions from experts with the primary goal of consensus building [27, 28]—to lead the expert panel in the development of a package of strategies that can be implemented by both health centers and CBOs. To maintain engagement and provide participants with varied formats to share perspectives, expert panel meetings included a mix of semi-structured large group discussions, small group breakout sessions and individual presentations from members of the group. Meetings focused sequentially on three broad topics that were defined a priori: (1) delineating key barriers to PrEP for LMSM in NYC, (2) identifying collaborative PrEP strategies that health centers and CBOs can implement in partnership; and (3) refining and finalizing a package of strategies.

During the first two meetings, the panel was asked to brainstorm a list of barriers to PrEP for LMSM and discuss their relative importance; after the discussion, they anonymously rated their importance on a Likert scale from 1 (not important at all) to 5 (extremely important). The third meeting included a visioning exercise in which panelists described ideal models of community-based PrEP delivery, followed by a brainstorming session on specific strategies to address the most relevant barriers identified by the panel in earlier sessions. In the fourth meeting, facilitators then led the group in a discussion of how strategies would fit within respective implementation contexts, after which panelists anonymously rated all identified strategies with respect to acceptability (“*How acceptable would each of these strategies be to you and the clients at your organization?*”), goodness of fit (“*How good a match would each of these strategies be within your organization?*”) and ease of implementation (“*How easy to implement would each of the following strategies be in your organization?*”). During the fifth and sixth meetings, facilitators presented these ratings back to the panel, followed by findings from focus groups with CBO clients about preferences for PrEP delivery, and asked them how themes identified by clients might influence strategies. Panelists were asked to consider all implementation strategies within the context of these quantitative and qualitative data, barriers that could impact implementation of the strategies, their potential impact on operations at each organization, and whether implementation over the next year was feasible. They then decided on a final package of strategies, which were anonymously rated using the validated acceptability of intervention measure (AIM), intervention appropriateness measure (IAM), and feasibility of intervention measure (FIM) [29]. For all rating exercises, we calculated mean scores and ranges for each item after each round of surveys was completed.

### Focus Groups with CBO Clients

We conducted three focus groups (one at each CBO) to obtain end-user input on PrEP delivery. Participants in focus groups provided verbal informed consent prior to enrollment in the study. With the support of CBO staff, we identified a convenience sample of clients at each organization meeting inclusion criteria and contacted them by phone to offer participation in a focus group discussion about PrEP. We developed a focus group guide informed by the CFIR and centered on three main topics: (1) dissemination of information about PrEP to communities; (2) dealing with structural barriers to obtaining PrEP; and (3) community empowerment. Participants were individuals aged ≥ 18 years who self-identified as Latino MSM, were HIV-negative, interested in or had used PrEP, and fluent in either English or Spanish. The focus groups were facilitated by two qualitative researchers. One focus group was conducted in-person at the CBO in Spanish and two were conducted online using Zoom in English. Focus group transcripts were professionally transcribed, translated.

The project PI (JR), community investigator (GB) and a research coordinator (EA) used rapid qualitative methods to analyze the data and obtain actionable and targeted results in a short timeframe and maintain scientific rigor [30]. We iteratively developed a standard template for summarizing focus group transcripts (Supplementary material), structured using the three topics covered in the focus group guide and subtopics that emerged from an initial reading of the transcripts. We then reviewed the transcripts and developed summaries using the template such that each transcript was summarized by two investigators. Summaries were then discussed with other team members to compare and combine templates, after which they were entered into a matrix that included columns for the topics and subtopics in the summary template and a row for data from each focus group. After populating the matrix with information from the summary templates, the three team members discussed and synthesized the information on each topic.

## Results

### Expert Panel Characteristics

**Fig. 1:**
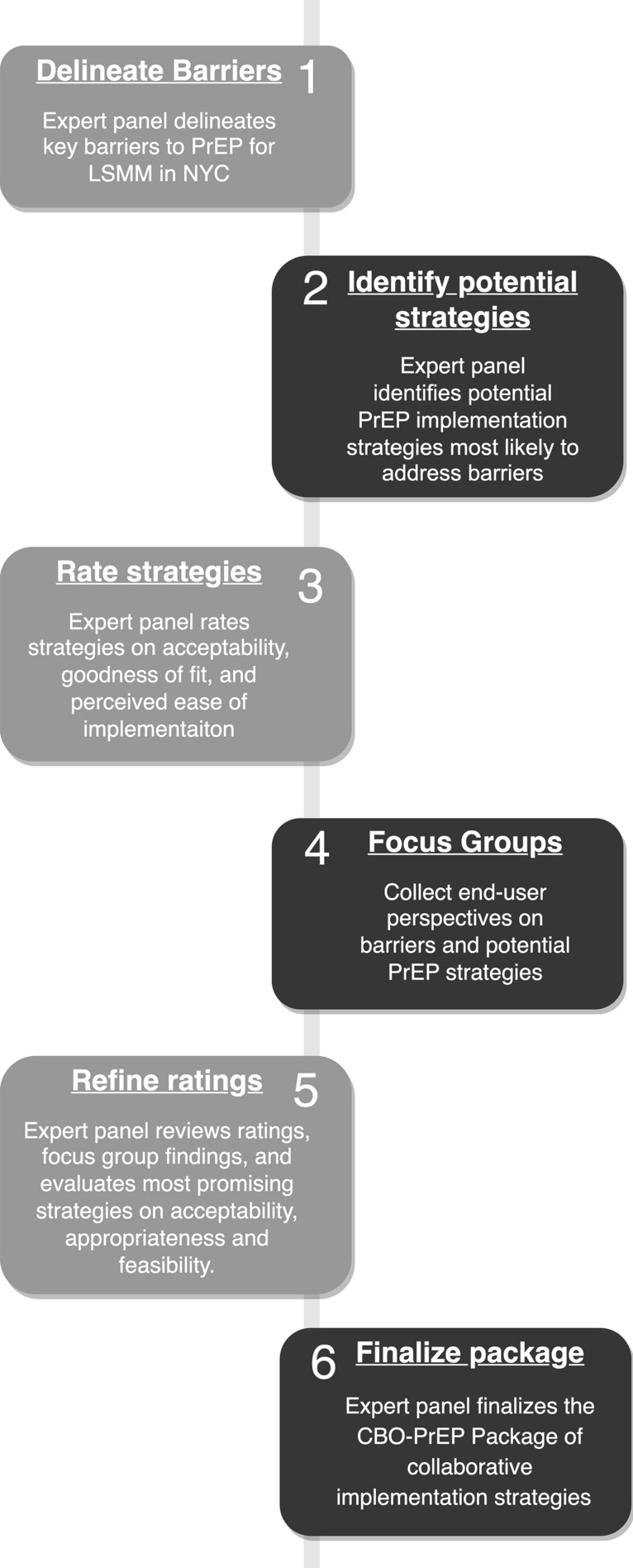
Modified Delphi process undertaken by the community expert panel to develop the CBO-PrEP package of implementation strategies

The expert panel consisted of eight individuals (two each from MPP and the three CBOs), including seven who identified as Latino/a/x, 5 men, 1 transgender woman, and 1 person who identified as genderqueer. Participants included CBO peer navigators, CBO program directors, a health center patient navigator, and a family medicine physician. Most participants (n = 6) were between 30 and 49 years old and had been working in their current position for an average of 5 years (range 1–9) and in HIV prevention for an average of 11 years (range 3–28). Between November 2021 and May 2022, the expert panel met six times, with meetings lasting two to three hours (Fig. 1).

### Delineating Key Barriers to PrEP for MSM

Initial meetings focused on identifying the most relevant barriers to PrEP for LMSM in NYC. Expert panelists identified barriers at the individual, institutional and intervention levels, compiling a list of 28 salient barriers. They were then asked to anonymously rate each of these barriers with respect to their importance, on a Likert scale from 1 to 5. The most important barriers included immigration-related fears about accessing care (mean score of 4.6), not wanting to take a daily medication (4.5), and concerns about PrEP side effects (4.4) and cost (4.4) (Table 1). The academic research team presented these ratings back to the expert panel and led a semi-structured discussion, ultimately grouping barriers into four thematic categories: (1) dealing with a complex and fragmented healthcare delivery system; (2) the process of getting connected to PrEP takes too long; (3) LMSM do not feeling empowered in healthcare settings; (4) concerns about PrEP medication.

**Table 1:** Relative importance of barriers identified by Community Expert Panel (N = 8)

Barrier	Mean score	IQR
Clients believe they cannot access services because of immigration status	4.6	(4.0, 5.0)
Clients do not want to take a medication everyday	4.5	(4.0, 5.0)
Clients have concerns over PrEP side effects	4.4	(4.0, 5.0)
Clients have more urgent or pressing needs than PrEP/HIV prevention	4.4	(4.0, 5.0)
Clients worry about the cost of PrEP	4.4	(4.0, 5.0)
Clients have low or limited English fluency	4.0	(3.0, 5.0)
Clients feel the health system is too complicated to navigate	3.9	(3.0, 4.3)
The process (all the paperwork, medical visit, labs, etc.) takes too long for clients	3.9	(3.0, 5.0)
The wait time for appointments is too long	3.9	(3.0, 4.3)
Clients do not see PrEP as a high priority	3.8	(3.8, 4.0)
Clients do not think they are at risk of HIV	3.8	(3.0, 4.0)
Clients do not know enough about what PrEP is or whether it could help them	3.8	(3.8, 4.0)
Clients feel that their doctors/providers don’t engage them enough after they first meet	3.8	(2.8, 5.0)
Clients find it too difficult to obtain necessary documents for PrEP navigation	3.8	(3.0, 4.3)
I lose the trust of clients because the process is too complicated	3.6	(3.0, 4.3)
Traveling to an in-person appointment takes too long	3.6	(3.0, 4.0)
Clients do not have enough free time to go to appointments	3.5	(3.0, 4.0)
Clients find the health setting is stigmatizing (because of homelessness)	3.5	(3.0, 4.3)
Clients find the health setting is stigmatizing (because of immigration status, language)	3.5	(2.0, 4.3)
Clients find the health setting is stigmatizing (because of sexual orientation, gender identity)	3.5	(2.0, 4.3)
Using technology is challenging for some clients	3.5	(2.8, 4.3)
Gathering paperwork to get clients connected to PrEP takes me too long	3.3	(2.8, 4.0)
The funds my organization receives limits what I can do	3.3	(2.5, 4.3)
It’s too hard for me/my organization to deal with the health system	3.1	(2.0, 4.0)
Clients feel that PrEP is too “medical”	3.0	(2.0, 3.5)
In my organization I don’t have enough time to navigate each client	3.0	(1.8, 4.3)
Clients do not have access to transportation	2.9	(1.8, 4.0)
Clients do not trust telemedicine	2.5	(1.8, 3.0)

Ranked using a Likert scale (1—not important at all; 5—extremely important)

*IQR* interquartile range, *PrEP* pre-exposure prophylaxis

### Identifying Collaborative Strategies That Health Centers and CBOs Can Implement in Partnership

Panelists were asked to envision a future where the barriers to PrEP they had previously identified were overcome. Panelists were then guided through a brainstorming activity to identify specific, concrete strategies health centers and CBOs could jointly implement to make this envisioned future possible and improve PrEP delivery for LMSM in NYC. This resulted in a list of 25 different strategies, which were then rated on acceptability, goodness of fit, and perceived ease of implementation (Table 2). Results were presented back to the panel for feedback and discussion; strategies were grouped into three main approaches: (1) strategies that improve communication between staff at health centers and CBOs; (2) low-barrier structural strategies that meet the preferences of LMSM; and (3) development and dissemination of culturally-tailored and locally-relevant outreach materials.

**Table 2:** Acceptability, appropriateness, and perceived feasibility of 25 strategies identified by a community expert panel (N = 8), ranked using Likert scales (1–5)

	Acceptability^a^		How good a match^b^		Ease of implementation^c^	
	Mean	IQR	Mean	IQR	Mean	IQR
Structural strategies						
Same-day telehealth appointments with a medical provider	4.9	(5, 5)	3.9	(3.3, 5)	3.1	(1.8, 5)
Simultaneous scheduling of an “intake” visit for documentation, followed by a medical visit	4.8	(4.8, 5)	4.4	(4, 5)	3.4	(2.8, 4)
Immediately providing a PrEP “starter pack” (30 days of pills) to new clients	4.4	(4, 5)	4	(3.8, 5)	3.1	(2, 3.5)
Clients do at-home HIV testing before/during telemedicine appointment	4.3	(3.8, 5)	4.6	(4.8, 5)	3.4	(2, 5)
Using conveniently-located commercial labs for PrEP-related lab tests	4.1	(4, 4.3)	4.5	(4, 5)	3.3	(2, 4.3)
Doing lab testing at the community-based organization	4.8	(4.8, 5)	4.4	(4, 5)	4.4	(4, 5)
Having a medical provider located at the community-based organization	4.3	(4, 5)	4.4	(4, 5)	3.8	(3.5, 5)
Scheduling PrEP appointments during evenings and weekends	4.8	(4.8, 5)	4.3	(3.8, 5)	3.3	(2.5, 4.3)
Delivering PrEP medications to clients’ homes	4.4	(4, 5)	4.6	(4, 5)	3.5	(2.8, 4.3)
Changing follow-up appointments to every 6 months (rather than every 3)	4.1	(3.8, 5)	3.8	(3, 5)	3	(2, 4)
Making PrEP available at urgent care centers	4.8	(4.8, 5)	3.8	(2.5, 5)	2.5	(2, 3)
Measuring tenofovir levels to provide clients with “feedback” on their PrEP adherence	4	(4, 4)	4	(3.8, 5)	3.1	(2.8, 4)
Communication strategies						
Developing a process to help clients gather needed documentation in advance of appointment	4.5	(4, 5)	4.6	(4, 5)	3.5	(3, 4)
Ensuring a warm hand off in real time between navigator and provider	4.4	(4, 5)	4.5	(4, 5)	4.3	(4, 5)
Use ally organizations/networks to provide important information about PrEP	4.5	(4, 5)	4.8	(4.8, 5)	4	(3.8, 5)
Training all healthcare providers (physicians, nurses, front desk, security) in cultural competency and inclusion	4.5	(4, 5)	4.6	(4.8, 5)	4.1	(3.8, 5)
CBO staff being able to directly schedule medical appointments for clients	4.6	(4, 5)	4.1	(3.8, 5)	3.3	(2, 4.3)
Outreach strategies						
Using social media to promote telemedicine as a way to get PrEP	4.3	(4, 5)	4.4	(4, 5)	4.3	(4, 5)
Creating educational materials/media that describe each step in the PrEP process	4.4	(4, 5)	4.6	(4, 5)	4.1	(3.8, 5)
Making sure that any outreach materials represent/include community members	4.5	(4, 5)	4.8	(4.8, 5)	4.1	(3.5, 5)
Using peers/promotores to address immigration-related PrEP concerns	4.8	(4.8, 5)	4.5	(4, 5)	4.1	(4, 5)
Using peers/promotores to provide important messaging about PrEP side effects, stigma and other concerns	4.4	(4, 5)	4.4	(4, 5)	3.9	(3.5, 5)
Using social media to provide important messaging about PrEP side effects, stigma and other concerns	4.3	(4, 5)	4.3	(4, 5)	3.9	(3.5, 5)
Using peers/promotores to promote telemedicine-based PrEP option	4.8	(4.8, 5)	4.9	(5, 5)	4.3	(3.8, 5)
Using social media to address immigration-related PrEP concerns	4	(3.8, 5)	4.4	(3.8, 5)	4.1	(3.8, 5)

*IQR* interquartile range, *PrEP* pre-exposure prophylaxis, *CBO* community-based organization

^a^Likert scale: 1—completely unacceptable; 5—completely acceptable

^b^Likert scale: 1—a terrible match; 5—a great match

^c^Likert scale: 1—extremely difficult to implement; 5—extremely easy to implement

### Understanding CBO Client Preferences and Perspectives

A total of three focus groups were held, one in-person (N = 8) and two virtually (N = 6 and N = 4). All participants in the focus groups lived in NYC and identified as Latino men; average age was 34 years, and 71% of those with data available reported ever using PrEP. Rapid qualitative analyses identified three themes: trusted sources of information, traditional health settings disempower LGBTQ persons, and LMSM have differing preferences for low-barrier PrEP-related care, which are summarized in Table 3 with illustrative quotes.

**Table 3:** Representative focus group quotes

Theme	Quotes
*Trusted sources of information*	“I’ve also heard about PrEP in young gay community youth centers. They’re always advertising it and just telling the youth to get on it.”
	“I only knew of what my friend [at the CBO] had told me about which I commented earlier. So, he is there and helps me with things. I ask him about this or that and he tells me that he could help me out or tells me what I can apply to. So, he gives me that trustworthy feeling that they can help me there and solve my issues. That’s why I began to take PrEP.”
	“It was also the recommendation of my doctor… that was my source of motivation.”
	“Something that helps us a lot is looking for people from our own community…. I went to [a community organization]…It’s a lot of trans girls. I didn’t have anything to do with that but that was my community. These people hear your stories. Those fears start to go away. Surrounding yourself with people of your community empowers you.”
*Traditional health settings disempower LGBTQ persons,*	“And [LMSM are] just scared, just because they just feel like they don’t want to just be there [in medical settings] overall. They feel like they’re not going to get help, whatsoever. They’re not going to be understood.”
	“I wasn’t comfortable sharing my status with anything of my sexual well-being with my doctors when I first had a primary doctor. It wasn’t until I found an LGBTQ doctor that I started to share about my sexual experiences.”
	“I went to this physician, and I just went for a regular STD test. And the moment I said that I was gay, and I also do anal sex—the look of disgust on her face was just, it was a cue to me that I’m not welcome here.”
	“It doesn’t matter if he is Latino or not, but someone of color who knows how someone like us can feel uncomfortable than with someone who is white. We don’t know how they are going to treat us. People of color, Indians, Latinos, Philippines, understand us. It’s more comfortable to speak to them.”
*LMSM have differing preferences for low-barrier PrEP-related care*	“I think it’s just better to have the doctor there, ask those questions that I don’t really want to feel comfortable in the telemedicine, because maybe when I’m doing a telemedicine appointment with them, there’s other people around that probably will hear me.”
	“I think that it is best on the phone, you know. When I go to the doctor, the time will be more or less but if you do it over the phone, you can go whenever. The doctor will call you when he is ready. Like that. It’s private too.”
	“I honestly prefer a private lab, because to me, you just get your work done and go. So, it’s way faster for me to do it at a private lab.”
	“I would prefer to come [to the CBO to get lab tests]…because I know the organization. I feel identified with the organization. If I were to go someplace else, well… Either way, there are always people who stare at you…”
	“There are curious people who like to ask, “Why are you here? What are you getting tested on? I see that you are healthy!” I don’t really have much patience for these questions so I would prefer a private lab.”

#### Trusted Sources of Information

Participants identified various sources of information, including social media, friends, sex partners, medical providers, and CBOs. Confidence was highest in information that came from trusted sources, particularly close friends, CBOs and medical providers. While social media was felt to be useful for widespread dissemination about PrEP, participants generally preferred the more nuanced and tailored information available through individual, one-on-one sources. Importantly, they described the choice to use PrEP as a process involving balancing the risks and benefits, often needing time as well as the encouragement of peers and service or medical providers to decide to pursue PrEP.

#### Traditional Health Settings Disempower LGBTQ Persons

Focus group participants described experiences of feeling uncomfortable or explicit discrimination in health settings. Others shared that finding a provider they felt comfortable with required a long process of shopping around, but that once they found this provider, they were willing to endure other logistical barriers. More broadly, participants strongly preferred medical centers that were specifically welcoming to LGBTQ patients and that employed staff and providers who shared lived experience with their patients. There was also support for disseminating information about PrEP in ways that were tailored to LGBTQ and immigrant communities.

#### LMSM Have Differing Preferences for Low-Barrier PrEP-Related Care

Focus group participants identified multiple individual and structural barriers to accessing PrEP, including lack of insurance and/or not knowing that coverage is available for individuals in NYC who want to access PrEP, immigration-related fears, discomfort in health settings, busy schedules that made it challenging to attending daytime appointments, transportation costs, and others. To this end, many supported low-barrier options such as evening appointments or getting required laboratory tests done at locations closer to their home or work. Opinions about telemedicine were mixed, with some participants enthusiastic about the prospect of spending less time getting to appointments and avoiding potentially stigmatizing experiences in clinics, and others worrying that it would be too impersonal or that others in their homes might overhear conversations with their provider. Across all three focus groups, participants very strongly endorsed the need for multiple options and individual choice around which low barrier options to use.

### Refining and Finalizing a Package of Strategies

The academic research team that analyzed qualitative transcripts presented focus group findings to the expert panel for review and discussion, along with prior ratings of strategies with respect to acceptability, goodness of fit, and perceived ease of implementation. In synthesizing these findings, the panel identified five general strategies to include in the final “CBO-PrEP Package:” (1) use of a web-based referral tool to track new PrEP referrals from CBOs; (2) use of a telemedicine option for new clients referred for PrEP; (3) use of geographically-convenient options for lab specimen collection for clients referred for PrEP; (4) health center and CBO collaboration to develop print and social media outreach materials providing information on PrEP, including the process of obtaining insurance coverage and making appointments with providers; and (5) holding regular implementation coaching meetings together with navigators at CBOs and health centers to identify and address patient and programmatic issues. These five strategies were then rated using the AIM, IAM and FIM (Table 4). Through ongoing collaboration with MPP, LCOA, VL and DT, and guidance from subsequent expert panel meetings, CBO-PrEP strategies were subsequently pilot tested, and we are currently studying their implementation at all four organizations.

**Table 4:** Acceptability, appropriateness and feasibility of final package of strategies, using AIM, IAM and FIM scales [27], rated by a community expert panel (N = 8)

Strategy	Acceptability		Appropriateness		Feasibility	
	Mean	IQR	Mean	IQR	Mean	IQR
Web-based tool for referring CBO clients to health center	4.1	(4.0, 4.3)	4.2	(4.2, 4.3)	4.2	(4.2, 4.2)
Telemedicine option for clients	4.7	(4.6, 4.8)	4.7	(4.6, 4.8)	4.7	(4.6, 4.8)
Use of geographically-convenient locations for lab specimen collection	4.6	(4.6, 4.7)	4.7	(4.6, 4.7)	4.3	(4.2, 4.4)
Co-development of outreach materials for CBO clients	4.7	(4.6, 4.8)	4.7	(4.6, 4.7)	4.8	(4.8, 4.8)
Regular meetings between navigation staff at health center and CBOs	4.4	(4.2, 4.5)	4.4	(4.4, 4.4)	4.4	(4.4, 4.5)

*AIM* acceptability of intervention measure (Likert score, 1–5), *IAM* intervention appropriateness measure (Likert score, 1–5), *FIM* feasibility of implementation measure (Likert score, 1–5)

## Discussion

There is consensus that community-centered approaches are critical to ending the HIV epidemic [17]. Although CBOs are frequently involved in navigating clients who are interested in or could benefit from PrEP to health centers, they are rarely included in designing and implementing PrEP-related care delivery. We undertook a systematic process with a group of community content experts representing CBO and health center perspectives to identify key barriers to PrEP and select a set of collaborative strategies that meet the needs and priorities of LMSM in NYC. This process resulted in a package of PrEP delivery strategies that leverage the trust of the LMSM community in CBOs, provide flexibility for clients, and reduce communication and administrative barriers between CBOs and health centers.

Both members of the community expert panel and focus group participants emphasized that messaging from CBOs is highly trusted by LMSM communities. Community stakeholders agreed that developing tailored messaging about PrEP—including culturally- and locally-relevant information about benefits, how to use it, and the process involved in obtaining it—was a highly acceptable, appropriate and feasible strategy. Panelists described how PrEP advertising targeting general populations—even messaging specifically directed to LMSM—often did not resonate with communities due to differences related to country of origin, immigration status, time spent in the U.S., gender identity, and other characteristics. A desire for tailored, culturally-focused messaging has been reported among LMSM in other settings [31, 32]. Because health center staff are familiar with the nuances of healthcare delivery (e.g. obtaining insurance, appointment scheduling) in their local setting and CBOs are highly familiar with and trusted by the communities they serve, developing outreach materials collaboratively was felt to be a strategy with high potential for success and may avoid pitfalls often associated with health outreach and education materials.

Through the community-engaged process utilized for this study, we identified a strong desire for flexible, tailored PrEP delivery strategies for LMSM. Two of the strategies selected by the expert panel—offering clients telemedicine appointments and the opportunity to have laboratory specimens collected in convenient locations—represent low-barrier, flexible options that address or circumvent barriers to conventional PrEP care, including transportation time and costs, competing priorities, and likely anticipated and enacted stigmas in physical health care settings. These represent feasible and widely scalable strategies: most health centers nationally have increased their telemedicine capacity because of the COVID-19 pandemic; similarly, there is fairly widespread availability of commercial laboratories (e.g. Quest, LabCorp) that may be more conveniently located than health centers [33, 34]. Such low-barrier strategies are effective for increasing PrEP engagement [35–37] and have been increasingly adopted in the setting of the COVID-19 pandemic [38, 39]. Importantly, both content experts and focus group participants indicated that these strategies would work for some but not all clients, and emphasized a need for tailored approaches that offer individual choice—findings that are consistent with the limited literature supporting individualized approaches to improve PrEP engagement among LMSM [31, 32].

Finally, the community expert panel supported approaches that would reduce communication barriers between CBOs and health centers. Specifically, the expert panel chose two strategies to improve coordination: a web-based referral tool for tracking of new PrEP referrals, and the use of regular monthly meetings to address patient navigation issues and facilitate implementation of other strategies. Prior qualitative studies with healthcare and CBO stakeholders highlighted the potential of health center-CBO partnerships to improve community health but also identified concerns about communication, absence of organizational infrastructure and lack of time for relationship-building as major barriers to these partnerships [40–42]. The work described in this manuscript moves beyond those findings by offering specific, tangible strategies that could address these barriers. Nonetheless, health centers and CBOs typically operate in separate and siloed regulatory environments, limiting effective collaboration. Advocating for policy changes that would enable more cooperative approaches (for example, facilitating reimbursements for effective health center-CBO partnerships) could have a significant impact on improving PrEP uptake for LMSM and other priority populations.

This study has several strengths, in particular the practical, community-engaged approach to establishing strategies for CBOs and health centers to collaborate in delivering PrEP to LMSM. Over a sustained period, we worked together with frontline staff from a large health center multiple CBOs serving LMSM communities in NYC to identify locally-relevant barriers to accessing PrEP, and then systematically identified and prioritized strategies to overcome these barriers. Importantly, this work not only included the perspectives of CBO clients (end-users), but had stakeholders reflect on and synthesize these data. Nonetheless, our study has some limitations. Findings may not be representative to LMSM in other settings that have fewer CBOs providing services to LMSM and where insurance and medication coverage may not be as robust. Moreover, the relatively small number of individuals participating in the community expert panel, from a select group of CBOs, may limit generalizability of our findings. Additionally, the demographic make-up of LMSM in NYC may not be reflective of other U.S. settings. Although we incorporated anonymous rating into our modified Delphi process, there may still have been social desirability bias present. The focus group guide was not pilot tested given short time interval available to undertake data collection, although we found that the guide worked well for promoting discussion and eliciting the needed information.

In conclusion, through a systematic and participatory approach with community content experts, we identified a set of highly acceptable, appropriate and likely feasible strategies that health centers and CBOs can collaboratively implement to improve PrEP engagement for LMSM in NYC. Engaging community members directly and meaningfully in the planning of HIV prevention and PrEP services is a critical step in identifying approaches with the potential to overcome barriers faced by LMSM. We are currently continuing this level of engagement through the implementation and evaluation of the package of strategies, to understand how these can best be operationalized in diverse CBO settings and to determine their impact on PrEP uptake and use. Ultimately, the processes described in this manuscript could be adapted to other populations and settings to enhance PrEP engagement and help to end the HIV epidemic.

### Supplementary Information

Below is the link to the electronic supplementary material.

Supplementary file1 (DOCX 16 KB)
