# Supplementary material for: Collaborative PrEP Implementation Strategies for Latino Men Who have Sex with Men: A Health Center-Community Consensus Process
Source: J Community Health. 2023 Aug 7;48(6):994–1003. doi: 10.1007/s10900-023-01266-w (PMC10558404; doi:10.1007/s10900-023-01266-w)
Supplement: Supplementary file 1 — Supplementary file1 (DOCX 16 KB) [file 10900_2023_1266_MOESM1_ESM.docx]

**CBO PrEP Focus Group Discussions**

**SUMMARY TEMPLATE**

FGD Number:

Summarizer:

**Theme 1: Information dissemination and access**

- **Question 1: What do you know about PrEP?**
- **Question 2: How did you learn about PrEP?**
- **What sources of information were most helpful?**
- **Question 3: What is the process for starting PrEP?**
- **Question 4: How did you decide to use PrEP or not?**

**Theme 2: Process: dealing with system barriers**

- **Question 1: Which of these are barriers/facilitators to access sexual health care and PrEP?**
- **Question 2: Which of these are you willing to accept?**
- **Question 3: Which one would you like to make easier?**
- **Question 4: What’s your preference for seeing a doctor? Telehealth or in-person**
- **Question 5: Where would you prefer to get you lab tests done?**

**Theme 3: Process: community empowerment**

- **Question 1: Have you felt uncomfortable/discriminated in healthcare settings?**
- **Question 2: What could be done to help you felt more uncomfortable and confident in accessing care?**
